# Prevalence of Intestinal Parasitic Infections among Patients of King Fahd Medical City in Riyadh Region, Saudi Arabia: A 5-Year Retrospective Study

**DOI:** 10.1155/2018/8076274

**Published:** 2018-07-26

**Authors:** Omar S. O. Amer, Esam S. Al-Malki, Mohamed I. Waly, Abdulaziz AlAgeel, Mahmoud Y. Lubbad

**Affiliations:** ^1^Medical Laboratory Sciences Department, College of Applied Medical Sciences, Majmaah University, 11952, Saudi Arabia; ^2^Zoology Department, Faculty of Science, Al-Azhar University (Assiut Branch), Assiut, Egypt; ^3^Medical Equipment Technology, College of Applied Medical Sciences, Majmaah University, 11952, Saudi Arabia; ^4^Microbiology Department, King Fahd Medical City, Ministry of Health, Riyadh, Saudi Arabia; ^5^Environmental and Occupational Health, Public Health Agency, Ministry of Health, Riyadh, Saudi Arabia

## Abstract

This study is a retrospective analysis of the recorded intestinal parasitic infections for in- and outpatients visiting King Fahd Medical City, Riyadh, Saudi Arabia, from 2013 to 2017. In this study, a total of 5987 in- and outpatient were examined for intestinal parasitic infection. 30 patients out of 5987 were infected with 6 species of intestinal parasites with prevalence rate 0.5%. These parasites were* Entamoeba histolytica* (P = 0.27%),* Cryptosporidium *sp. (P = 0.1%),* Giardia lamblia* (P = 0.07%),* Trichuris trichiura* (P = 0.03%),* Hymenolepis nana* (P = 0.02%), and* Chilomastix mesnili* (P = 0.02%). The prevalence of infection in both males and females was 0.38% and 0.58%, respectively. Also, the prevalence of infection in different years and age groups as well as different seasons was provided. Intestinal parasitic infections are still a public health problem in Riyadh region, Saudi Arabia. Updating the epidemiologic survey of these parasites at regular intervals using the appropriate statistical methods is necessary to develop effective prevention and control strategies.

## 1. Introduction

The intestinal parasitic infections in developing countries are considered the main cause of public health problem [1, 2]. The recent studies revealed that around 30% of the total population in the world is infected with intestinal parasites [3, 4]. The prevalence of intestinal parasitic infections is considerably varied in the different regions of the world. It depends on so many factors such as geographic and socioeconomic factors, relatively humid areas, poverty, malnutrition, personal and community hygiene, high population density, unavailability of potable water and low health status, and poor sanitary facilities [5–7]. These factors give the optimum conditions for the growth and transmission and increase the probability of exposure to intestinal parasites [5–7]. Also, it has been affected by the used diagnostic methods and the number of the investigated stool samples [8]. The major tracks for transmission of intestinal parasites are the contamination of food or drinking water or personal contact via fecal-oral route [9]. As well known, Saudi Arabia is considered one of the largest destinations of expatriate workers, particularly the food handlers and catering staff, from different countries such as Bangladesh, Philippine, India, Indonesia, Pakistan, Sri Lanka, and Egypt. All of these countries are known to be endemic with many diseases including those caused by intestinal parasites [8]. Many studies in different regions of Saudi Arabia monitored a high prevalence of infection with intestinal parasites among specific populations such as food handlers (23%), school children (33.8%), expatriates (14.9% to 55%), and Saudi and Non-Saudi patients attending hospitals (39.7% to 77.1%) [8, 10–18]. As per the available literature, there are no updated surveys for intestinal parasitic infections that were carried out in Riyadh region except that of [19]. In that research, a retrospective study was conducted for intestinal parasitic infections of military personnel during the period from 2010 to 2014. Hence, this study aims to produce an updated report on the intestinal parasitic infections from 2013 to 2017 using the data records of King Fahd Medical City in Riyadh by applying the appropriate statistical methods.

## 2. Materials and Methods

The present study is a retrospective analysis of 5987 in- and outpatient stool samples reports from the King Fahd Medical City, Riyadh, KSA, during the period from 2013 to 2017 for intestinal parasitic infections.

### 2.1. Samples Collection and Examination

Samples were collected in sterile plastic containers, carefully labeled and transported to the Microbiology lab. The stool specimens were examined macroscopically for detecting the colour, consistency, the presence of mucous and blood, and any adult worms. On the other hand, the direct microscopic examination using normal saline and iodine was used to detect the protozoan parasites. For coccidian parasites, the permanent stained smears using modified Ziehl-Neelsen and modified trichrome stains were performed.

### 2.2. Data Collection

The datasets of stool samples examination for a total 5987 in- and outpatients during the period from 2013 to 2017 were collected from the hospital information system database department based on prior permission from the administration officials of the hospital.

### 2.3. Data Analysis

The collected data were yearly classified to determine the intestinal parasitic infections per years. The prevalence of infection for all and individual parasites per year, season, sex, and age groups was calculated and presented in tables accordingly. All statistical analysis was done using SPSS program version number (24) and the normality of collected data was tested using the Kolmogorov-Smirnov (KS) test. The nonparametric test, Chi-square test, was applied to check the association between the used variables (years, seasons, sex, and age groups) and the intestinal parasitic infections at confidence level 95%.

## 3. Results

The datasets of a total of 5987 in- and outpatients examined stool samples were collected, classified, and analysed using the nonparametric Chi-square test. Among those examined samples, there were 3616 (60.4%) females and 2371 (39.6%) males. Out of 5987 in- and outpatients who had been examined during the period of study (2013 – 2017), 30 patients were infected (P = 0.5%) with 6 species of intestinal parasites. These recorded parasites were* Entamoeba histolytica*,* Giardia lamblia*,* Cryptosporidium parvum*,* Chilomastix mesnili*,* Trichuris trichiura*, and* Hymenolepis nana*.

### 3.1. Prevalence of Parasitic Infections per Years and Seasons

Overall, the prevalence of protozoan infections (0.45%) was higher than the helminth infections (0.05%).* Entamoeba histolytica* (0.27%) and* Cryptosporidium parvum* (0.1%) were the common identified intestinal protozoan parasites. Only two helminth parasites were recovered:* Trichuris trichiura* (0.03%) and* Hymenolepis nana *(0.02%). The prevalence of parasitic infections during the period of study (2013 – 2017) is shown in Table 1. In terms of the prevalence of infection with season, it was higher in fall (0.79%) and summer (0.63%) seasons and lower in winter (0.25%) and spring season (0.35%) (Table 2).

### 3.2. Prevalence of Parasitic Infection in Both Males and Females

Overall, the prevalence of parasitic infection was higher (0.58%) in females than males (0.38%).* Entamoeba histolytica* was the most prevalent protozoan in both males (0.30%) and females (0.25%); however the helminth parasites were recovered from females only (Table 3).

### 3.3. Prevalence of Parasitic Infection in Different Age Groups

Overall, the prevalence of parasitic infection was higher (0.89%) in the age group (11 – 20 years) and lower (0.17%) in the age group (31 – 40 years); however no infections were recorded in age group (51 – 60 years) (Table 4).

## 4. Discussion

It is well known that the intestinal parasitic infections are in close relation to the poor sanitary habits and lack access to safe potable water and improper hygiene. It is worth mentioning that KFMCH is accessible only for Saudi citizens besides limited number of foreigners who work for the government. The current study revealed that 0.5% of examined patients were infected with 6 species of intestinal parasites. The previous studies of stool analysis surveys, in Saudi Arabia and many other countries, have revealed a wide range of variation in the prevalence of intestinal parasites. The prevalence was 13.3% in India [20], 32.0 – 41.5% in Palestine [21], 8.8% in Iran [22], 57.9% in Iraq [23], 10.2% in Qatar [24], 64.4% in Sudan [25], 7.7% in UAE [26], and 58.7% in Yemen [27]. However in KSA, the prevalence rate was 27.2% in Al-Ahsa [28], 47.01% in Jeddah [29], 6.2% in Makkah [30], 8.4% in Tabuk [31], and 2.3 – 39.7% in Riyadh [15, 32–34]. The much lower prevalence of infection during this study, compared to the previous studies particularly in Riyadh region, could be explained in one hand to the fact that most of the examined patients were Saudi citizens and most of them urban dwellers with a high socioeconomic status. On the other hand, it may be attributed to the general improvement in health services and sanitary conditions and increase awareness and health education in the Kingdom [32, 34–37]. The most common parasite in this study was* Entamoeba histolytica* and this finding is consistent with many previous reports outside and inside Saudi Arabia [26, 28, 30, 31, 38–41]. However, it disagrees with some others outside and inside the Kingdom, particularly those conducted in Riyadh region [8, 15, 19, 23, 27, 32, 34, 42]. This finding could be attributed to the susceptibility for infection by* Entamoeba histolytica* and/or the availability of the sources of infection by this parasite. Regarding the helminth infection, the current study revealed low prevalence of these parasites, compared to the protozoans' infection. This result is consistent with the previously reported surveys in many different regions inside the Kingdom, particularly in Riyadh region [8, 15, 28, 30–32, 34, 40, 42]. However, this finding disagrees with the most recent survey conducted in Riyadh region [19]. The similarity between the aforementioned finding and those reported surveys in many regions inside the Kingdom, including the Riyadh region, may reflect the consistency in environmental factors, socioeconomic conditions, and health status of these regions, while the dissimilarity could be attributed to the unfavourable ecological and sociocultural factors that influence the survival and transmission of soil transmitted helminths.

The prevalence of intestinal parasitic infections was slightly higher in females (0.58%) than males (0.38%); however this difference is not statistically significant (*P* = 0.569). The higher infection in females can be justified by considering the females are more exposed to the infective stages of parasitic infection due to the nature of the chores they perform in the house and their life style. This finding is in agreement with some previously reported surveys inside and outside the Kingdom [8, 40, 43]. Nevertheless, this result is not compatible with some other reports outside and inside the Saudi Arabia [23, 26, 31]. This study also revealed that the higher prevalence (0.89%) was recorded among 11–20-year age group, while the lower prevalence was recorded among 31–40-year age group. Otherwise, there is no infection recorded among 51–60- and above 70-year age groups. Statistically, there is no significant difference detected among the different age groups at* P* = 0.656. This finding disagrees with some previous studies outside and inside Saudi Arabia [15, 19, 23, 28, 30, 32, 34, 44, 45]. The high prevalence among 11–20-year age group could be attributed to the close contact with the contaminated environment through their tendency to spend most of their times in playing outside their houses and to eat the fast foods from the restaurants. The relationship between the intestinal parasitic infection and seasonality was studied in the current survey. The higher prevalence of infection was recorded in fall and summer seasons (0.79% and 0.63%), while the lower prevalence of infection was recorded in winter and spring (0.25% and 0.35%), respectively. Statistically, there is no significant difference detected in prevalence of infection among the different seasons (*P* = 0.546). The slight difference in prevalence of infection between fall and summer seasons could be attributed to the difference in examined number of patients in the two seasons. Our finding in the current study is nearly consistent with the previous studies which had been conducted in Qassim region [46] and [19] in Riyadh region. Also, in those studies the authors attributed the high prevalence of infection during the summer season to many factors affecting the transmission of parasitic infections. These factors were related to the human exposure to valley water collections during outdoor activities in summer time and the favourable ecological conditions such as temperature and humidity. The current authors, to large extent, accept the explanation as reasons for increasing the prevalence of infection during the summer season.

## 5. Conclusion

Overall, the low prevalence of intestinal parasitic infections in the current study reveals the improvement in living conditions and hygiene besides the combined efforts of the healthcare authorities in Riyadh region.* Entamoeba histolytica* was the most common intestinal parasite in the examined patients and the infection in females was higher than males. Moreover, 11–20-year age group was the most affected group and the higher infection was in summer and fall seasons. The food and drink safety and health education should be increased to raise the awareness of society about intestinal parasitic diseases. Therefore, it is important to keep a constant epidemiological surveillance through periodical surveys and improving the healthcare to resolve the problem of parasitic infections.

## Figures and Tables

**Table 1:** Prevalence of intestinal parasitic infection during the period of study (2013 - 2017).

**Type of parasite**	**2013**		**2014**		**2015 **		**2016 **		**2017 **	
	**No.**	**P **(%)	**No.**	**P **(%)	**No.**	**P **(%)	**No.**	**P **(%)	**No.**	**P **(%)
*Entamoeba histolytica*	7	0.52%	2	0.17%	0	0.00%	4	0.29%	3	0.05%

*Cryptosporidium parvum*	6	0.44%	0	0.00%	0	0.00%	0	0.00%	0	0.00%

*Giardia lamblia*	2	0.15%	0	0.00%	0	0.00%	1	0.07%	1	0.02%

*Trichuris trichiura*	1	0.07%	0	0.00%	1	0.07%	0	0.00%	0	0.00%

*Chilomastix mesnili*	0	0.00%	0	0.00%	0	0.00%	1	0.07%	0	0.00%

*Hymenolepis nana*	0	0.00%	0	0.00%	0	0.00%	1	0.07%	0	0.00%

**Total infected **	**16**	**1.18**%	**2**	**0.17**%	**1**	**0.07**%	**7**	**0.51**%	**4**	**0.07**%

**Total examined**	**1354**		**1158**		**1521**		**1373**		**581**	

**Table 2:** Prevalence of intestinal parasitic infection in different seasons during the study (2013 – 2017).

**Type of parasite**	**Spring**		**Summer **		**Fall **		**Winter **	
	**No.**	**P **(%)	**No.**	**P **(%)	**No.**	**P **(%)	**No.**	**P **(%)
*Entamoeba histolytica*	2	0.14%	5	0.39%	7	0.43%	2	0.12%

*Cryptosporidium parvum*	2	0.14%	1	0.08%	2	0.12%	1	0.06%

*Giardia lamblia*	0	0.00%	2	0.16%	2	0.12%	0	0.00%

*Trichuris trichiura*	1	0.07%	0	0.00%	1	0.06%	0	0.00%

*Chilomastix mesnili*	0	0.00%	0	0.00%	1	0.06%	0	0.00%

*Hymenolepis nana*	0	0.00%	0	0.00%	0	0.00%	1	0.06%

Total infected	5	0.35%	8	0.63%	13	0.79%	4	0.25%

Total examined	1446		1280		1643		1618	

**Table 3:** Prevalence of intestinal parasitic infection in both males and females during the study (2013 – 2017).

**Type of parasite**	**Female**		**Male**	
	**No.**	**Prevalence**	**No.**	**Prevalence.**
*Entamoeba histolytica*	9	0.25%	7	0.30%

*Cryptosporidium parvum*	5	0.14%	1	0.04%

*Giardia lamblia*	3	0.08%	1	0.04%

*Trichuris trichiura*	2	0.06%	0	0.00%

*Chilomastix mesnili*	1	0.03%	0	0.00%

*Hymenolepis nana*	1	0.03%	0	0.00%

Total infected	21	0.58%	9	0.38%

Total examined	3616		2371	

**Table 4:** Prevalence of intestinal parasitic infection in different age group during the study (2013 – 2017).

**Type of parasite**	**Age**		**Age **		**Age**		**Age **		**Age **		**Age**		**Age**		**Age **		**Age **		**Age**		**Age **	
	**0 - 10 y**		**11 - 20 y**		**21 - 30 y**		**31 - 40 y**		**41 - 50 y**		**51 – 60 y**		**61 - 70 y**		**71 - 80 y**		**81 - 90 y**		**91 - 100 y**		**101- 110 y**	
	**No.**	**P **(%)	**No.**	**P **(%)	**No.**	**P **(%)	**No.**	**P **(%)	**No.**	**P **(%)	**No.**	**P **(%)	**No.**	**P **(%)	**No.**	**P **(%)	**No.**	**P **(%)	**No.**	**P **(%)	**No.**	**P **(%)
*E. histolytica*	2	0.10%	4	0.89%	5	0.46%	2	0.17%	2	0.39%	0	0.00%	1	0.47%	0	0.00%	0	0.00%	0	0.00%	0	0.00%

*C. parvum*	5	0.26%	0	0.00%	0	0.00%	0	0.00%	1	0.19%	0	0.00%	0	0.00%	0	0.00%	0	0.00%	0	0.00%	0	0.00%

*G. lamblia*	3	0.15%	0	0.00%	1	0.09%	0	0.00%	0	0.00%	0	0.00%	0	0.00%	0	0.00%	0	0.00%	0	0.00%	0	0.00%

*T. trichiura*	2	0.10%	0	0.00%	0	0.00%	0	0.00%	0	0.00%	0	0.00%	0	0.00%	0	0.00%	0	0.00%	0	0.00%	0	0.00%

*Ch. mesnili*	0	0.00%	0	0.00%	1	0.09%	0	0.00%	0	0.00%	0	0.00%	0	0.00%	0	0.00%	0	0.00%	0	0.00%	0	0.00%

*H. nana*	1	0.05%	0	0.00%	0	0.00%	0	0.00%	0	0.00%	0	0.00%	0	0.00%	0	0.00%	0	0.00%	0	0.00%	0	0.00%

Total infected	13	0.67%	4	0.89%	7	0.64%	2	0.17%	3	0.58%	0	0.00%	1	0.47%	0	0.00%	0	0.00%	0	0.00%	0	0.00%

Total examined	1947		449		1094		1191		518		329		211		160		77		9		2	

## Data Availability

The data used to support the findings of this study are available from the corresponding author upon request.
